# Milk Production Responses and Digestibility of Dairy Buffaloes (*Bubalus bubalis*) Partially Supplemented with Forage Rape (*Brassica napus*) Silage Replacing Corn Silage

**DOI:** 10.3390/ani11102931

**Published:** 2021-10-10

**Authors:** Di Zhou, Mohamed Abdelrahman, Xinxin Zhang, Shuai Yang, Jing Yuan, Zhigao An, Kaifeng Niu, Yanxia Gao, Jianguo Li, Bo Wang, Guangsheng Zhou, Liguo Yang, Guohua Hua

**Affiliations:** 1 Key Laboratory of Animal Genetics, Breeding and Reproduction, Ministry of Education, College of Animal Science and Technology, Huazhong Agricultural University, Wuhan 430070, China; hzau_judy_213@163.com (D.Z.); mohamed.asad@agr.au.edu.eg (M.A.); zxx1144795936@163.com (X.Z.); ys1105216413@163.com (S.Y.); 17806243701@163.com (J.Y.); anzhigao95@foxmail.com (Z.A.); nkf_19930806@163.com (K.N.); 2 Animal Production Department, Faculty of Agriculture, Assuit University, Asyut 71515, Egypt; 3 College of Animal Science and Technology, Hebei Agricultural University, Baoding 071000, China; yxgaohebau@126.com (Y.G.); jgli@hebau.edu.cn (J.L.); 4 Key Laboratory of Crop Ecophysiology and Farming System in the Middle Reaches of the Yangtze River, College of Plant Science and Technology, Huazhong Agricultural University, Wuhan 430070, China; wangbo@mail.hzau.edu.cn (B.W.); zhougs@mail.hzau.edu.cn (G.Z.); 5 Hubei Province Buffalo Engineering Center, Wuhan 430070, China

**Keywords:** forage rape silage, buffalo, digestibility, milk composition, rumen fermentation

## Abstract

**Simple Summary:**

To develop alternative silage resources, we employed buffaloes as an animal model to evaluate the possibility and effects of forage rape silage in the dairy buffalo diet. We comprehensively assessed the nutrition value of forage rape silage by the apparent total-tract digestibility, rumen fermentation characteristics, blood metabolism and milk composition of lactating buffaloes. Our current results showed that the inclusion of forage rape silage in diets improved the milk quality, such as milk protein, milk fat, and total solid percentage. Furthermore, partial supplementation of forage rape silage also promotes buffaloes’ dry matter intake. These may be related to the favorable physiological and metabolic changes induced by the forage rape silage. Thus, our current data show the applicability of forage rape silage as a good feed resource for ruminants.

**Abstract:**

Worldwide, silage is considered the main component in dairy animal diets; however, this portion is mainly dominated by corn silage, which raises availability challenges in some agricultural production systems. The present study evaluated a partial replacement of corn silage with forage rape silage (FRS) and its effect on feed intake, nutrient digestibility, rumen fermentation, milk production, and blood metabolites in buffalo. Thirty-six lactating buffaloes were randomly assigned to four different groups, according to supplementation of FRS (only corn silage, FRS_0_) or with 15% (FRS_15_), 25% (FRS_25_), and 35% (FRS_35_) of forage rape silage instead of corn silage. The results showed that, compared to corn silage, forage rape silage has a lower carbohydrate but a higher protein concentration. The buffalo intake of dry matter and organic matter were improved linearly with the FRS increasing in the diet. The apparent total-tract digestibility (ATTD) of dry matter, organic matter, nitrogen, neutral detergent fiber, and acid detergent fiber also increased by the FRS supplementation compared with FRS_0_. Conversely, FRS supplementation decreased the propionic, butyric, and valeric acid contents and increased the acetic:propionic ratio and microbial protein content. Furthermore, FRS inclusion led to a significantly higher milk urea and non-fat milk solid content, higher blood glucose, total globulins, blood urea nitrogen, and lower blood high-density lipoprotein. These results suggested that FRS has high a nutritional value and digestibility, is a good feed resource, and showed favorable effects when supplemented with dairy buffalo ration.

## 1. Introduction

Recently, dairy ruminant production systems, especially buffalo, have been concerned with using high nutritive-yielding crops as a complementary forage source [1,2,3,4,5]. The brassica family, especially forage rape (*Brassica napus*), attracted valuable ruminant forage. Forage rape is considered a kind of high-quality green-forage source that is strongly preferred for its cultivating and feeding characteristics. In addition to the growing ability under the water-limited condition and high growth rate in the cold season [6], forage rape is considered as a high-yielding dry matter crop [7] and is easily intercropped with legumes [8]. Most importantly, forage rape is distinguished with its high nutritional value, especially the high metabolizable energy level (2.8~3.0 Mcal/kg dry matter (DM)), besides the readily fermentable carbohydrates, high crude protein (CP) (160 to 200 g/kg DM), and low fiber content [9,10]. Interestingly, feeding the brassica family and forage rape could also reduce methane emissions per unit of DM intake in ruminants, promoting its inclusion in ruminant diets [8,9,10,11]

Despite the high nutritional characteristics of brassica-family forages [9,10], the precise effects on dairy animal performance are much debated. In the recent literature, contradicting findings on milk production efficiency have emerged; for example, researchers reported the negative impact of feeding summer turnips (*Brassica rapa*) on milk production [11]. However, others mentioned that supplementing dairy cows with forage rape could improve milk production [12].

Questions have been raised about the sustainability of the on-farm fresh forage rape usage due to some limitations. One concern is that the low fiber content may induce a rapid rumen pH drop, affecting nutrient metabolism and other dietary components’ digestibility. Furthermore, the presence of high levels of antinutritional factors, such as the relatively high level of nitrates, increases the incidence of nitrites toxicity and even death of the animal [13]. In addition, secondary compounds (e.g., S-methyl-cysteine sulphoxide (SMCO) and glucosinolates) that mainly exist in brassicas in higher concentrations compared to other forage types also raised concerns. Likewise, the higher content of nitrogen (N) in forage rape may affect animal health through excessive rumen ammonia absorption [14].

It was previously known that the ensiling process could improve the forage quality, especially by inhibiting antinutritional factors. Unfortunately, studies that discussed ensilaged forage rape are limited; moreover, the investigations that assess dairy buffalo utilization for forage rape silage (FRS) are also scarce. Our previous study successfully introduced a forage rape silage method [14]. Therefore, this study aimed to evaluate the potentials of FRS inclusion on dairy buffalo performance, the ruminal fermentation pattern, and milk production response.

## 2. Materials and Methods

### 2.1. Animals, Diets, and Experiment Design

Thirty-six Mediterranea×Nili-Ravi hybrid lactating buffaloes were utilized for feeding experiments at the Hubei Jinniu Buffalo Farm (Hubei, China). Animals were randomly assigned to 4 groups according to initial milk yield, days in milking (DIM), lactation number, and bodyweight (BW) (mean ± SD), which were 5.1 ± 1.2 kg/day, 101 ± 76 days, 2.3 ± 1.1, 605 ± 86 kg, respectively. The diet was offered twice (at 600 and 1500 h) daily, while all animals had free access to water.

Forage rape (*Brassica napus* L.) cultivar (‘Huayouza 62’) were sown in late September 2018 and harvested in early April 2019. The plants were cut at the final flowering stage, air-dried for 72 h until the dry matter was over 30%. After harvesting, plants were chopped to a 3~5-centimeter length using a lawn mower, then mixed with corn meal (processed from corn kernels) (Northeast Treasury Corn Jilin, Jilin, China), brown sugar (from Fengtang Zhikang Brown-Sugar Factory, Chaozhou, China), and a bacterial inoculants mixture for the silage process. The bacterial mixture contained solid and liquid bacterial agents; the solid agents were Lactobacillus Plantarum, Lactobacillus acidophilus, Lactobacillus brucei, Lactobacillus casei, cellulase, effective live bacteria ≥4 × 10^9^ CFU/g. However, the liquid agents were Lactobacillus plantarum, Lactobacillus acidophilus, Pediococcus pentosaceus, organic acids, small molecule peptides, effective live bacteria ≥2 × 10 ^9^ CFU/g (Shandong Zhuohua Biological Company, Jinan, China). At Hubei Jinniu Buffalo Farm, the chopped forage rape was spread and compacted in a silage bunker layer by layer (each layer about a 20~30-centimeter height), then evenly sprayed with the mixture of the bacterial agents and corn meal in each layer, finally sealing. Each ton of chopped forage rape was treated by a bacterial inoculants’ mixture containing 2 L of water and 0.2 kg of brown sugar, with a water temperature adjusted to between 30 and 37 °C; this was then well mixed with 25 g of a solid bacterial agent, 250 mL of a liquid bacterial agent and 10 kg of corn flour.

The experiment provided four total mixed ration(TMR) diets, and its ratio of roughage to concentrate was 65:35 (calculated on dry matter basis), and the forage rape silage to corn silage (FRS:CS ratio) (on a fresh matter (FM) basis) in the diet was 0:100 (FRS_0_) and 15:85 (FRS_15_), 25:75 (FRS_25_), and 35:65 (FRS_35_), respectively. All diets contain (on a dry matter basis) 15% straw, 15% corn meal, 7% soybean meal, and 15% yeast culture. The chemical composition of each ingredient is listed in Table 1, and Table 2 provides the nutritive value characteristics of the four experimental diets. The study lasted for 73 days, the first 10 days as a feeding-adaptation period and a 63-day experimental period.

### 2.2. Sampling and Chemical Analyses

#### 2.2.1. Feed

Feed samples of the four experimental were collected at 9 a.m. on the 3rd, 24th, and 45th day of the experimental period. All feeding samples were dried at 65 °C for 48 h to determine the initial moisture content. Then, samples were grinded using a multifunctional pulverizer (Old Bank Boou Hardware Factory, Zhejiang, China) and passed through a 40-mesh sieve (manufactured by Tianxing Wusi Yarn Sieve Factory in Shangyu City, Zhejiang, China) to be stored at −20 °C for further analysis. The dry matter (DM), crude ash (CA), crude protein (CP), ether extract (EE), neutral detergent fiber (NDF), acid detergent fiber (ADF), acid detergent lignin (ADL), and acid insoluble ash (AIA) of feed were determined according to the Association of Official Analytical Chemists (1995) [15], and by described methods [16,17] and concentrations of NDF and ADF, both inclusive of residual ash, and ADL, were determined with the ANKOM Filter Bag Method. Both Heat-stable α-amylase and sodium sulphite were added during NDF extraction [18,19]. Water-soluble carbohydrates (WSC) concentration was determined using the anthrone sulphate method [20].

#### 2.2.2. Feces and Rumen Fluid Sampling and Analysis

Randomly selected three buffaloes with similar body conditions were reared individually for the apparent total-tract digestibility (ATTD) analysis. Each experimental diet included a 7-day adaptation period and a three-day experimental period (fed once a day, weighed diet, and the refusals). Fecal samples were collected at 9:00 and 17:00 every day, 600 g per buffalo (based on FM), and all fecal in one day to be mixed. Mixed fecal samples for every day were divided into two parts. One was added with 20 mL of 10% H_2_SO_4_ per 100 g of fresh fecal for fixing nitrogen [18], and then the another was stored directly at −20 °C for further analysis. Thawed fecal samples were dried and grinded to pass through a 40-mesh sieve. Concentrations of NDF and ADF in fecal were analyzed as described above. In addition, fresh fecal samples were analyzed for DM and CA, using the methods mentioned above, and nitrogen (N) using the Kjeldahl method.

Rumen fluid was collected by a ruminal content sampling tube (MDW16, Sichuan, China) from three buffaloes of each group 2 h after the morning diet on the 2nd and 62nd days. The collected rumen fluid was immediately filtered with a sterile 4-layer gauze, and the filtrate was divided into 10-milliliter Eppendorf tubes to be treated [21]. First, the rumen fluid pH was measured immediately using a pH meter (FE-20-FiveEasy PlusTM, Mettler Toledo Instruments Co., Ltd., Shanghai, China). Then, 10 mL of rumen fluid and 0.1 mL of 6 mol/L hydrochloric acid were mixed to fix ammonia-N. After that, 5 mL of rumen fluid was centrifuged (10,000× *g*, 10 min) using a refrigerated centrifuge (Thermo election corporation). Next, 1.5 mL of its supernatant was taken to be mixed with 0.15 mL of metaphosphoric acid (25%), shake homogenized, left to stand for 30 min, and centrifuged again (10,000× *g*, 15 min); the supernatant was taken for the determination of volatile fatty acid (VFA) [22]. Fresh and treated samples were stored at −20 °C for further analysis. The ammonia-N concentration was determined using Phenol-sodium hypochlorite colorimetry [23], the concentration of microbial protein (MCP) was determined using the Coomassie brilliant blue method, and gas chromatography was used for the analysis of VFA concentrations [24].

#### 2.2.3. Milk

Milk samples were taken at 500 and 1500 h every day, and the milk yield was recorded. In addition, milk samples were collected from each buffalo once per day, alternating morning and afternoon milking from the 15th and 16th day of each period. Milk samples were 1:1 mixed and conserved with preservative (0.2 g of bronopol solution/40 mL of milk), kept refrigerated at 4 °C, and afterwards analyzed for fat, total protein, lactose, and urea at an official milk control laboratory (Hubei Provincial Animal husbandry Bureau, Wuhan, China), using Fourier transform infrared spectroscopy (MilkoScan 7RM, FOSS Analytical, Hillerød, Denmark) [25].

#### 2.2.4. Blood

On the 5th and 58th day of the experiment, six buffaloes of each group were randomly selected for 20 mL of blood to be collected from the neck vein using heparinized vacuum tubes 2 h after the morning feeding. Each sample was mixed gently and centrifuged at 3000 r/min for 15 min at room temperature (low-speed centrifuge, SCIL0GEX, Beijing, China). Plasma was recovered, transferred to plastic vials, and frozen at −20 °C for analysis of biochemical blood parameters. The total protein (TP), blood urea nitrogen (BUN), glucose (Glu), total cholesterol (TC), triglycerides (TG), high-density lipoprotein (HDL), and low-density lipoprotein (LDL) were detected using a biochemical analyzer (automatic biochemical analyzer, BS-420, Shenzhen Mindray, Guangdong, China). In addition, alanine aminotransferase (ALT), aspartate aminotransferase (AST), glutamyltransferase (GGT), and lactate dehydrogenase (LD) were tested by kits (Nanjing Jiancheng, Nanjing Jiancheng Institute of Biological Engineering Limited, Nanjing, China) [26].

### 2.3. Calculations and Statistical Analyses

#### 2.3.1. Calculations

The dry matter intake (DMI) calculation formula was DMI = feed intake (kg/day) × DM content of the feed (%). Assuming a fecal recovery of acid-insoluble ash (AIA) is 100% [27,28], the fecal OM content was calculated by measuring the crude ash content in the feces. Apparent Total-tract digestibility (ATTD) for DM, OM, NDF, and ADF was calculated in a group as ATTD% = 100 × (1 − (nutrient in Fecal × AIA in diet)/(nutrient in diet × AIA in Fecal)).

#### 2.3.2. Statistical Analyses

All statistical analyses were analyzed using SAS 9.4 (SAS Institute, Cary, NC, USA, 2017). Data were normally distributed and homoscedastic. The following statistical mixed model (repeated measure) was used for all variables (except for ATTD, and rumen fluid index):

Y_ijk_ = μ + τ_i_ + δ_ij_ + t_k_ + (τ × t)_ik_ + ε_ijk_ (1)

where Y_ijk_ is the dependent variable, µ is the overall mean, τ_i_ is the fixed effect of dietary treatment i, t_k_ is the random effect of time, (τ × t)_ik_ is the interaction effect of group and time, δ_ij_ is the random effect of covariance between repeated measures in an individual, and ε_ijk_ is the random error of the variance between the measures within the individual. ATTD and rumen fluid index were analyzed according to the following model (one-way ANOVA):

Y_ij_ = μ + τ_i_ + ε_ij_ (2)

where Y_ijk_ is the dependent variable, µ is the overall mean, τ_i_ is the fixed effect of dietary treatment i, and ε_ij_ is the random residual error. Data were analyzed using an ANOVA way to identify treatment effects. Additionally, multiple contrast tests related to Tukey were conducted for treatment comparisons. Additionally, orthogonal polynomial contrasts were used to test for linear and quadratic effects of RS levels on response variables. All reported values are least significant means (LSM), and significance was declared at *p* < 0.05.

## 3. Result

### 3.1. Feed Intake and Apparent Total-Tract Digestibility

Forage rape supplementation effects on a buffalo’s feed intake and apparent total-tract digestibility (ATTD) are shown in Table 3. Increasing the proportion of FRS in the diet linearly raised the DMI from 9.33 to 10.47 kg/day (*p* < 0.0001), and the OM intake from 8.00 to 8.86 kg/day (*p* < 0.0001), respectively. Supplementation of 25% (FRS_25_) and 35% (FRS_35_) FRS significantly improved the DMI by 16.9 and 12.2% compared with that in the FRS_0_ group. Supplementation of 25 and 35% of FRS also significantly increased the OM intake by 14.6 and 10.8%, respectively.

The FRS_15_ and FRS_25_ diets significantly promoted apparent total-tract DM, OM, N, NDF, and ADF digestibility (Table 3). However, the FRS_35_ treatment showed a deteriorated apparent total-tract DM and OM digestibility (*p* < 0.05). Moreover, the apparent total-tract NDF and ADF digestibility of the FRS_15_ and FRS_25_ diets were significantly enhanced compared with FRS_0_ and FRS_35_. Meanwhile, N digestibility also significantly increased in the FRS_15_ and FRS_25_ treatments compared to the FRS_0_ and FRS_35_ diets.

### 3.2. Rumen Fermentation Characteristics

It was observed that pH and ammonia-N levels were not affected by FRS inclusion; however, the FRS_25_ and FRS_35_ diets showed a 16–17% lower ammonia nitrogen concentration than detected in the FRS_0_ and FRS_15_ groups. Moreover, the microbial protein content (5.85 mg/mL) of the FRS_25_ diet is significantly (*p* < 0.0001) higher than FRS_0_, FRS_15_, and FRS_35_ (2.52, 3.25, and 2.05 mg/mL, respectively). Additionally, the FRS_25_ diet dramatically increased the microbial protein content (5.85 mg/mL); however, the highest FRS level (FRS_35_) recorded the lowest microbial protein content (2.05 mg/mL). Interestingly, further rumen analysis revealed that the ruminal C2:C4 fatty acids profile (acetic, propionic, butyric, and isobutyric) followed a parallel pattern, whereas FRS supplementation tends to decrease the C2:C4 fatty acid level. Similarly, C5 fatty acids (valeric and Isovaleric acid) and caproic acid (C6) showed the same trend, whereas FRS_15_ was the lowest concentrations for valeric, Isovaleric, and caproic acids (1.38, 1.02, and 0.219 mol/L, respectively) (Table 4).

### 3.3. Milk Yield and Composition

The daily milk yield in buffaloes was not influenced by forage rage silage supplementation. However, FRS numerically improved some milk components, such as the concentrations of milk protein, milk fat, total solids (TS), non-fat milk solid (SNF), casein, saturated fatty acids (SFA), and monounsaturated fatty acids (MUFA) (*p* < 0.05), when compared with that in the FRS_0_ group (Table 5). The milk lactose concentration remained unchanged with the supplementation of FRS (*p* = 0.0734). The milk urea concentration showed a significant response (*p* = 0.0385) to FRS levels, and the FRS_35_ diet resulted in the highest concentration compared to FRS_0_, FRS_15_, and FRS_25_. The SNF concentrations in FRS_25_ and FRS_35_ treatments were higher than FRS_0_ and FRS_15_.

### 3.4. Blood Parameters

As showin in Table 6, the FRS_15_ and FRS_25_ diets significantly increased blood glucose, total protein, total globulins, and blood urea nitrogen concentration compared with the other treatments. The FRS_15_ and FRS_25_ diets significantly (*p* < 0.0001) decreased the blood HDL concentration compared to FRS_0_ and FRS_35_. The FRS_15_ diet resulted in the highest total blood protein content (130.21 g/L) among all the treatments. No differences were observed for total cholesterol (TC), LDL, and liver enzymes (ALT, AST, LD, and GGT) between the different diets.

## 4. Discussion

As mentioned in previous studies, brassicas forages contain anti-nutritional sulphur compounds, such as S-methyl cysteine sulphoxide and glucosinolate, which are highly associated with a strong and distinct flavor that affects feed palatability [6] and milk composition, and causes manufacturing complications [11,29]. The current results showed that FRS supplementation improved the DMI, contrary to the previous studies that reported feeding kale (*Brassica oleracea*) decreased the DMI when offered solely to dairy cows [30]. Although Keim et al. [31] reported that fresh forage rape did not alter dairy cows’ DMI, this difference is due to fresh form compared to ensilaged forage rape. Additionally, the negative impact of glucosinolate was diluted through the ensilaging process through the microbial inoculants, which enhanced glucosinolate microbial hydrolysis and supported ruminal flora degradation activity [32,33].

In the FRS_0_ group, the apparent total-tract fiber-fractions digestibility was the lowest compared to the FRS diets, which resulted from the diet’s low NDF and ADF content accompanied by a high level of water-soluble carbohydrates. This latter factor depressed the fiber fractions’ digestibility, improved the ruminal VFA content and decreased the ruminal pH, as stated in previous studies [34,35], which can be observed from the short-chain fatty acids (SCFA) content and pH level in the present study. On the other hand, consistent with Lambert et al. [36], the FRS_15_ and FRS_25_ diets recorded a higher digestibility for DM, OM, and NDF due to the dietary balance between readily fermentable carbohydrates and low content hemicellulose and cellulose in brassica forage [9]. However, the FRS_35_ level seems to be a suppressor for DM, OM, and fiber digestibility consistent with its high NDF content that dampens nutrient digestibility [37], besides the high concentration of S-methyl cysteine sulphoxide, which is mainly found in brassica-fed stock [6]. Additionally, the ATTD of N increased due to the increasing dietary CP content between the groups; these findings are supported by Schulz et al. [38].

Ruminal pH values were in the normal range for healthy buffalo [39] and in agreement with Kaur and Garcia findings, which stated that FR inclusion could not alter pH levels [40]. As it is known, the role of NDF can promote chewing activity and increase the salivary-buffer flow to the rumen [41,42]; this explanation is also integrated by previous studies that highlighted the role of high-NDF digestion in raising the ruminal pH above 6.3 [43]. Although we observed the ruminal ammonia N decline in FRS_25_ and FRS_35_, it was not significantly affected by FRS inclusion, which agrees with previous studies [40,44]. The FRS_25_ diet resulted in the lowest ammonia N, because of the microbial recapture for volatile N in the rumen and utilizing it in microbial growth. Consequently, the FRS_25_ diet led to the highest level of microbial protein synthesis due to the dietary balance between C and N that increased the microbial protein content. On the other hand, the ruminal VFA decreased by FRS inclusion compared to the FRS_0_ diet, attributable to FRS_0_ dietary characteristics concerning the highest content in water-soluble carbohydrate and the lowest in fiber fractions (NDF and ADF), which also explains the highest ruminal VFA values for the FRS_0_ diet. Correspondingly, the ruminal VFA content followed a rising pattern from FRS_15_ to FRS_35_, and that molar proportion of VFAs fractions increased the acetic:propionic ratio; also, the SCFA (C2:C4) Medium-chain fatty acids (MCFAs)was increasingly similar to [45], which responded to an increasing DMI in the FRS groups.

In agreement with Seguel et al.’s [46] results, the milk yield was not affected by FRS inclusion; this resulted from the higher acetic:propionic ratio that directs the energy utilization toward butterfat at the expense of milk yield [47]. This statement is likely to agree with the numerical improvement pattern in fat content among the FRS groups. Similarly, milk urea showed a positive correlation with FRS inclusion, partly explained by the increased dietary CP content that promotes surplus amino acid catabolism, which alters the urea pool [2,3,48]. The significant rise in milk solids not fat (SNF) was associated with higher milk protein synthesis; this finding is similar to Rugoho [49] report’s about feeding dairy cows with kale (*Brassica oleracea*) and its positive effect on milk solids content. Both the milk SFA and MUFA improvement in the FRS groups responded to the improvement in the C2:C3 and C4:C5 ruminal fatty acids profile.

In the present study, blood glucose correlated positively with the ruminal propionate content, which stimulates insulin and alters the glucose level [50], which disagreed with previous reports [51] due to age and species difference. Furthermore, in concordance with a previous study [52], blood globulins in FRS_15_ and FRS_25_ groups were significantly higher, which can be indicative of the glucosinolate intake that increases the liver size and hepatic activity [53], leading to more liver globulins production that increases total globulin and total protein. However, the contradicted values for LDL and TC in the FRS_15_ and FRS_25_ groups were similar to previous studies that reported the concentration ratio’s role in the expression extent of the glucosinolate effect [54]. Additionally, the dietary CP intake is the furthermost reported reason for increasing blood urea levels as described by [2,51,55].

## 5. Conclusions

This study confirmed the potential of FRS inclusion in early–mid lactating buffalo diets without a detrimental effect on milk production performance and the ruminal fermentation pattern, which may expand the usage of this forage type in buffalo diets by recommending FRS_15_ and FRS_25_ levels. Thus, the current results will strengthen our knowledge about the efficient utilization of forage rape and other brassicas forage types. However, this study cannot answer the nutritional value and feasibility of the ensilaging process, which can minimize the identified limitation for forage rape. Thus, further comparative investigations are needed, especially for examining the ensilaging effect on physiological and ruminal kinetics.

## Figures and Tables

**Table 1:** Chemical composition of feed ingredients.

Parameter (%DM)	Forage Rape Silage	Corn Silage	Straw	Corn Meal	Soybean Meal	DDGS
Dry matter (DM)	30.77	21.67	85.32	89.64	90.61	89.94
Organic Matter (OM)	88.58	92.7	87.16	98.08	93.33	90.48
CP	11.74	8.74	4.11	13.05	42.81	20.36
Ether extract	2.94	2.58	1.00	3.52	2.27	4.64
Water-soluble carbohydrates	2.77	7	6.28	12.68	12.91	7.93
Neutral Detergent Fiber (NDF)	51.13	54.32	51.9	5.72	12.8	34.26
Acid Detergent fiber (ADF)	34.4	28.67	31.51	0.88	7.47	21.84
Acid Detergent Lignin (ADL)	5.63	2.87	1.59	ND^1^	ND	5.6

^1^ ND = not determined.

**Table 2:** Ingredients and chemical composition of the experimental diets.

Item	Diets ^1^			
	FRS_0_	FRS_15_	FRS_25_	FRS_35_
Ingredient (%DM)				
Forage rape silage	0	9.87	16.21	22.36
Corn silage	46.20	38.03	32.84	27.79
Straw	15.52	15.03	14.70	14.38
Corn meal	15.62	15.13	14.80	14.48
Soybean meal	7.03	6.81	6.66	6.51
DDGS	15.62	15.13	14.80	14.48
Brick lick ^2^	-	-	-	-
Chemical composition				
OM, % of DM	92.37	90.97	90.69	90.19
CP, % of DM	12.25	13.17	13.52	14.16
Ether extract, % of DM	2.17	2.31	2.98	3.71
Water Soluble Carbohydrate (WSC), % of DM	7.06	5.61	3.71	5.61
NDF, % of DM	33.52	35.37	37.09	37.68
ADF, % of DM	20.05	23.06	24.22	24.33

^1^ Experimental diets were composed of forage and concentrates (83:17), with targeted levels of forage rape silage (FRS):corn silage (CS) = 0:100, 15:85, 25:75, and 35:65 on an FM basis. ^2^ A lick brick weighs 5 kg and consists of 98% salt, 25 mg of calcium, 250 mg of phosphorus, 1000 mg of magnesium, 20 mg of selenium, 150 mg of copper, 50 mg of cobalt, 500 mg of iron, and 200 mg of manganese in each group of four lick bricks.

**Table 3:** Feed intake and apparent total-tract digestibility in lactating buffaloes fed the four experimental diets.

Item	Diets ^1^				SEM	*p*-Value ^2^		
	FRS_0_	FRS_15_	FRS_25_	FRS_35_		Diet	L	Q
Intake, kg/day								
DM	9.33 ^b^	9.65 ^b^	10.91 ^a^	10.47 ^a^	0.1793	<0.0001	<0.0001	0.0173
OM	8.00 ^b^	8.07 ^b^	9.17 ^a^	8.86 ^a^	0.1508	0.0001	<0.0001	0.1070
ATTD, %								
DM	59.31 ^c^	65.83 ^a^	62.54 ^b^	54.48 ^d^	0.6811	<0.0001	0.0004	<0.0001
OM	62.25 ^b^	68.61 ^a^	65.26 ^ab^	57.30 ^c^	0.7404	<0.0001	0.0006	<0.0001
N	61.53 ^c^	64.17 ^ab^	64.90 ^a^	62.10 ^bc^	0.5723	0.0087	0.3646	0.0014
NDF	42.21 ^c^	53.53 ^ab^	55.79 ^a^	44.61 ^bc^	1.9912	0.0032	0.3199	0.0005
ADF	35.02 ^b^	52.29 ^a^	54.72 ^a^	36.33 ^b^	2.3448	0.0005	0.5608	<0.0001

^a–d^ LSM within the same row with different superscripts differ (*p* < 0.05). ^1^ Experimental diets were composed of forage and concentrates (83:17), with targeted levels of forage rape silage (FRS):corn silage (CS) = 0:100, 15:85, 25:75, and 35:65 on an FM basis. ^2^ L = linear effect; Q = quadratic effect.

**Table 4:** Rumen fermentation characteristics in lactating buffaloes fed the four experimental diets.

Item	Diets ^1^				SEM	*p*-Value ^2^		
	FRS_0_	FRS_15_	FRS_25_	FRS_35_		Diet	L	Q
pH	6.92	7.00	6.98	7.05	0.0476	0.4122	0.1561	0.9019
Ammonia nitrogen, mg/dL	6.51	6.83	5.39	5.43	0.3633	0.3157	0.1656	0.8443
MCP, mg/mL	2.52 ^b^	3.25 ^b^	5.85 ^a^	2.05 ^b^	0.4408	<0.0001	0.4804	<0.0001
Acetic acid, mmol/L	28.07	20.28	24.51	24.83	2.0031	0.1432	0.5719	0.0717
Propionic acid, mmol/L	23.30 ^a^	14.98 ^b^	19.72 ^ab^	20.35 ^ab^	1.6139	0.0398	0.6014	0.0202
Acetic/Propionic	1.20 ^b^	1.36 ^a^	1.24 ^ab^	1.22 ^b^	0.0287	0.0215	0.6402	0.0122
Butyric acid, mmol/L	21.65 ^a^	14.36 ^b^	17.24 ^ab^	20.01 ^ab^	1.5380	0.0428	0.7821	0.0087
Isobutyric acid, mmol/L	1.78	1.41	1.66	1.72	0.1120	0.2010	0.8890	0.0850
Valeric acid, mmol/L	2.47 ^a^	1.38 ^b^	2.10 ^ab^	2.27 ^a^	0.1873	0.0149	0.8974	0.0071
Isovaleric acid, mmol/L	1.34	1.02	1.27	1.16	0.0826	0.1062	0.4770	0.2326
Caproic acid, mmol/L	0.342	0.219	0.324	0.347	0.0461	0.2630	0.5872	0.1462

^a,b^ LSM within the same row with different superscripts differ (*p* < 0.05). ^1^ Experimental diets were composed of forage and concentrates (83:17), with targeted levels of forage rape silage (FRS):corn silage (CS) = 0:100, 15:85, 25:75, and 35:65 on an FM basis. ^2^ L = linear effect; Q = quadratic effect.

**Table 5:** Milk yield and milk composition in lactating buffaloes fed the four experimental diets.

Parameter	Diets ^1^				SEM	*p*-Value ^2^		
	FRS_0_	FRS_15_	FRS_25_	FRS_35_		Diet	L	Q
Milk, kg/day	5.11	5.30	5.26	4.80	0.9565	0.9534	0.7518	0.6380
Protein, %	4.08	4.12	4.26	4.66	0.1712	0.1121	0.0190	0.5727
Fat, %	7.34	7.55	7.98	8.55	0.5318	0.1335	0.0221	0.6403
Lactose, %	5.59	5.26	5.59	5.46	0.1389	0.0734	0.8861	0.3219
Urea, mg/dL	20.34 ^ab^	17.50 ^b^	19.01 ^ab^	21.26 ^a^	1.3034	0.0385	0.3080	0.0095
TS, %	17.47	17.23	18.36	19.04	0.6984	0.0532	0.0126	0.3582
SNF, %	10.08 ^ab^	9.74 ^b^	10.34 ^a^	10.39 ^a^	0.2171	0.0202	0.0321	0.2139
Casein, %	2.68	2.70	2.89	3.00	0.1457	0.1080	0.0187	0.6555
SFA, %	4.81	4.93	5.14	5.55	0.3744	0.2354	0.0494	0.5921
MUFA, %	2.29	2.35	2.56	2.69	0.1821	0.1210	0.0199	0.7481

^a,b^ LSM within the same row with different superscripts differ (*p* < 0.05). ^1^ Experimental diets were composed of forage and concentrates (83:17), with targeted levels of forage rape silage (FRS):corn silage (CS) = 0:100, 15:85, 25:75, and 35:65 on an FM basis. ^2^ L = linear effect; Q = quadratic effect.

**Table 6:** Blood parameters in lactating buffaloes fed the four experimental diets.

Parameter	Diets ^1^				SEM	*p*-Value ^2^		
	FRS_0_	FRS_15_	FRS_25_	FRS_35_		Diet	L	Q
Glu, mmol/L	4.52 ^b^	6.29 ^a^	5.91 ^a^	4.69 ^b^	0.4877	0.0030	0.9362	0.0003
TC, mmol/L	7.40	7.96	7.81	7.17	0.3264	0.0893	0.4299	0.0170
TP, g/L	99.92 ^b^	130.21 ^a^	104.35 ^b^	97.85 ^b^	8.6030	0.0041	0.2525	0.0067
TG, mmol/L	2.57 ^b^	3.44 ^a^	3.32 ^a^	2.57 ^b^	0.1497	<0.0001	0.8065	<0.0001
LDL, mmol/L	3.25	3.53	3.39	3.30	0.1756	0.4168	0.9823	0.1509
HDL, mmol/L	1.43 ^a^	1.02 ^b^	1.04 ^b^	1.41 ^a^	0.0900	<0.0001	0.8873	<0.0001
BUN, mmol/L	6.65 ^b^	7.17 ^ab^	7.47 ^a^	6.20 ^b^	0.2885	0.0014	0.2670	0.0003
ALT, U/L	39.37	39.57	34.21	38.04	3.2667	0.3499	0.3758	0.4412
AST, U/L	28.79	26.92	22.40	32.71	6.6176	0.4894	0.7331	0.2078
LD, U/L	721.32	616.73	692.31	659.92	54.448	0.2838	0.5352	0.3596
GGT, U/L	45.12	45.41	50.10	44.38	4.0831	0.5024	0.8496	0.3106

^a,b^ LSM within the same row with different superscripts differ (*p* < 0.05). ^1^ Experimental diets were composed of forage and concentrates (83:17), with targeted levels of forage rape silage (FRS):corn silage (CS) = 0:100, 15:85, 25:75, and 35:65 on an FM basis. ^2^ L = linear effect; Q = quadratic effect.

## Data Availability

The datasets used and/or analyzed during the current study are available from the corresponding author.
